# Spatiotemporal Characterization of Changes in the Respiratory Tract and the Nervous System, Including the Eyes in SARS-CoV-2-Infected K18-hACE2 Mice

**DOI:** 10.3390/v17070963

**Published:** 2025-07-09

**Authors:** Malgorzata Rosiak, Tom Schreiner, Georg Beythien, Eva Leitzen, Anastasiya Ulianytska, Lisa Allnoch, Kathrin Becker, Lukas M. Michaely, Sandra Lockow, Sabrina Clever, Christian Meyer zu Natrup, Asisa Volz, Wolfgang Baumgärtner, Malgorzata Ciurkiewicz, Kirsten Hülskötter, Katharina M. Gregor

**Affiliations:** 1 Department of Pathology, University of Veterinary Medicine Hannover, 30159 Hannover, Germany; 2 Center for Systems Neuroscience, University of Veterinary Medicine Hannover Foundation, 30159 Hannover, Germany; 3 Institute of Virology, University of Veterinary Medicine Hannover, 30159 Hannover, Germany

**Keywords:** BavPat1, central nervous system, CNS, COVID-19, K18-hACE2 mice, neuropathology, ocular manifestation, SARS-CoV-2, severe acute respiratory syndrome coronavirus 2, spatiotemporal distribution, upper and lower respiratory tract

## Abstract

Severe acute respiratory syndrome coronavirus 2 (SARS-CoV-2), the causative agent of coronavirus disease 2019 (COVID-19), is known to affect multiple organ systems, including the respiratory tract and nervous and ocular systems. This retrospective study aimed to characterize the spatiotemporal distribution of viral antigen and associated pathological changes in the nose, lungs, brain, and eyes of K18-hACE2 mice intranasally infected with SARS-CoV-2. Using histology and immunohistochemistry, tissues were examined at 3, 6, and 7/8 days post-infection (dpi). In addition, lung and brain tissues were analyzed by means of RT-qPCR to determine viral RNA titers. Viral antigen was most pronounced in the nose, brain, and lung at 3, 6, and 7/8 dpi, respectively, whereas viral antigen was detected at 6 and 7/8 dpi in the retina. Quantitative PCR confirmed increasing viral RNA levels in both lung and brain, peaking at 7/8 dpi. Nasal and lung inflammation mirrored viral antigen distribution and localization. In the brain, the predominantly basal viral spread correlated with lymphohistiocytic meningoencephalitis, neuronal vacuolation, and altered neurofilament immunoreactivity. Retinal ganglion cells showed viral antigen expression without associated lesions. Microglial activation was evident in both the optic chiasm and the brain. These findings highlight the K18-hACE2 model’s utility for studying extrapulmonary SARS-CoV-2 pathogenesis. Understanding the temporal and spatial dynamics of viral spread enhances insights into SARS-CoV-2 neurotropism and its clinical manifestations.

## 1. Introduction

Severe acute respiratory syndrome coronavirus 2 (SARS-CoV-2), the etiologic agent of coronavirus disease 2019 (COVID-19), has had a far-reaching impact on multiple organ systems in humans. The course of infection varies greatly, ranging from asymptomatic to fatal, with the virus affecting not only the respiratory tract but also the cardiovascular, gastrointestinal, urogenital, and nervous systems. While respiratory symptoms predominate, systemic and neurological disease manifestations have emerged as significant clinical features [1]. Ocular manifestations, although less common, have been observed in approximately 2–32% of patients [2,3], with even higher prevalence being reported in adolescents [4].

Animal models such as non-human primates, hamsters [5,6,7,8,9], and in particular a transgenic mouse model, which expresses human angiotensin-converting enzyme 2 under cytokeratin 18 promoter (K18-hACE2), have provided key insights into the pathogenesis of SARS-CoV-2. Originally developed to investigate severe acute respiratory syndrome virus infection [10], K18-hACE2 mice have been instrumental in understanding the severe and systemic effects of SARS-CoV-2 [11], particularly its neuroinvasive properties [10,12,13,14,15].

Following intranasal infection, SARS-CoV-2 replicates within both the nasal respiratory epithelium (RE) and the olfactory neuroepithelium (OE) of K18-hACE2 mice, leading to anosmia and inflammation in the nasal cavity within the first week post-infection, similar to COVID-19 in humans [12,16,17,18]. Pathomorphological lesions in the upper respiratory tract include mild multifocal neutrophilic rhinitis, segmental necrosis with co-localized intracytoplasmic SARS-CoV-2 protein and RNA in the RE, while intraepithelial viral antigen and RNA, lacking inflammation, are evident in the OE. These lesions are typically transient, resolving within one week of infection [12].

Virus spread extends to the central nervous system (CNS) [12,14,18,19], resulting in a range of clinical manifestations, including weight loss, lethargy, as well as neurological dysfunctions (e.g., tremor, proprioceptive defects, abnormal gait) [14,15,17]. The route of neuroinvasion in this animal model is suspected to be via trans-synaptic spread through cranial nerves, including the olfactory or vagus nerve, as well as hematogenous entry or a combination of both [20,21,22,23,24,25]. Peak of inflammatory changes and viral protein spread was reported between 6 and 8 days post-infection (dpi) [15,25,26] with lesions present throughout the cerebrum, but absent or minimal in the cerebellar cortex and adjacent white matter [12,25,26]. In the literature, CNS findings described in K18-hACE2 mice include lymphohistiocytic [12,14,16,19,25,26] and neutrophilic [14,16] meningoencephalitis with microgliosis [12,14,25,26] and occasionally astrogliosis [12]. In addition, thrombi [15,25], perivascular hemorrhage [19,25], vasculitis [25], as well as neuronal spongiosis [12,16,25], myelin sheath vacuolation in the white matter [25], and neuronal [12,16,19] and glial [16,26] cell death are reported.

Although rare, ocular manifestations can sometimes be the first or sole sign of COVID-19 that may arise at any point during the disease or even after recovery [27]. Therefore, ocular involvement of SARS-CoV-2 has gained increasing attention. In humans, the presence of SARS-CoV-2 RNA in conjunctival swabs and tear samples from infected patients has raised questions about the role of the eye in the transmission and pathogenesis of the virus [27,28]. The presence of the key entry receptors angiotensin-converting enzyme receptor 2 (ACE2) and transmembrane protease serine subtype 2 (TMPRSS2) in human and murine ocular tissues [29,30,31,32] along with studies showing successful ocular inoculations in several animal models [33,34,35,36] suggest that infection can occur via the conjunctival route. In addition to these findings, SARS-CoV-2 RNA has been detected in various ocular structures, including the human cornea [37], scleral fibroblasts, retina, and oligodendrocytes in the optic nerve [38,39,40]. Moreover, the virus has been shown to be capable of replicating in vitro within conjunctival epithelial cells [41] and retinal ganglion cells [42]. Clinical findings in COVID-19 patients have revealed ocular manifestations such as conjunctivitis or retinal microvasculopathy, hemorrhages and edema, retinal axonopathies (“cotton-wool spots”), and retinal infarcts [28,40,43], indicating that the eye may serve as a site for both viral pathology and systemic vascular dysfunction. In animals, coronaviruses have long been recognized for their ability to cause ocular diseases, as seen in felines and mice [44]. Despite reports of retinal involvement, detailed histopathological studies of ocular tissues in COVID-19 animal models remain scarce. Animal models, such as hamsters and mice, have shown that SARS-CoV-2 can infect ocular tissues, leading to inflammation and potential visual impairment, mimicking some of the clinical findings in human COVID-19 patients [35,36].

Understanding the neurological and ocular implications of SARS-CoV-2 is crucial for both human and veterinary medicine, particularly for individuals and animals in high-risk environments. Further research is needed to elucidate the exact mechanisms of viral entry, replication, and transmission through the tissues, bridging the knowledge gap between respiratory and non-respiratory manifestations caused by this pathogen. Therefore, the aim of this retrospective study is to further explore the spatiotemporal distribution of lesions in the nose, lungs, brain, and eyes of SARS-CoV-2-infected K18-hACE2 mice. This could ultimately contribute to better prevention and management strategies for both respiratory and non-respiratory complications of COVID-19 in humans.

## 2. Materials and Methods

### 2.1. Animals

All experiments were performed in compliance with the ARRIVE (Animal Research: Reporting of In Vivo Experiments) guidelines, the European Directive 2010/63/EU, and the national Animal Welfare Act, with the approval of the Lower Saxony State Office for Consumer Protection and Food Safety (33.19-42502-04-20/3440).

Male and female B6.Cg-Tg(K18-ACE2)2Prlmn/J (K18-hACE2) transgenic mice were reared at the Institute of Biochemistry (University of Veterinary Medicine Hannover, Hannover, Germany) and kept in isolated ventilated cages under BSL3 conditions at the Research Center for Emerging Infections and Zoonoses (RIZ, University of Veterinary Medicine Hannover, Hannover, Germany). K18-hACE2 mice received water ad libitum and were fed with standard rodent diet (ssniff Spezialdiäten GmbH, Soest, Germany).

### 2.2. Study Design

Tissues from 8-weeks-old male and female K18-hACE2 mice (*n* = 28) infected with SARS-CoV-2 were investigated with regard to the presence and distribution of SARS-CoV-2 antigen and associated lesions in nasal (*n* = 27) as well as lung tissues (*n* = 27), brain (*n* = 28), globes (*n* = 26) and optic chiasms (*n* = 5–7). Mice were infected intranasally with SARS-CoV-2 (BavPat1/2020 strain, 5 × 10^4^ TCID50, *n* = 21) or mock-infected with phosphate-buffered saline (PBS, *n* = 7). Infected animals were sacrificed at 3 (*n* = 8), 6 (*n* = 8), 7 (*n* = 4), and 8 (*n* = 1) days post-infection (dpi).

### 2.3. Histopathology

Brain tissue was immersed in 10% neutral buffered formalin for 72 h, whereas globes and nasal tissue were fixed for 3 weeks. In addition, nasal tissue was decalcified in SOLVAGREEN^®^ (#6484.3, Carl Roth GmbH & Co. KG, Karlsruhe, Germany) for 7 days. Subsequently, tissue specimens were embedded in paraffin wax, cut into 2–4 μm-thick sagittal serial sections, and mounted on glass slides for histological evaluation and immunohistological analysis. Tissue sections were then stained with hematoxylin and eosin (HE) using an automated slide stainer (Leica ST 4040; Leica Biosystems, Nussloch, Germany). In addition, globes were stained with cresyl fast violet and alcian blue as described previously [45].

### 2.4. Immunohistochemistry

Immunohistochemistry was performed to detect the distribution and spread of SARS-CoV-2 as well as associated tissue changes, using either the avidin-biotin-peroxidase complex (#VEC-PK6100, VECTASTAIN^®^ Elite^®^ ABC Kit, Vector Laboratories, Biozol Diagnostica Vetrieb GmbH, Eching, Germany) method or the Dako EnVision+ polymer system (#K4001, Dako EnVision+ System-HRP Labeled Polymer, Agilent Technologies Inc., Santa Clara, CA, USA) according to the manufacturer’s protocol. Detailed information about the antibodies used, including dilutions and antigen retrieval, is given in Table S1. Tissue sections were deparaffinized in Roticlear^®^ (#A538.3, Carl Roth GmbH & Co. KG, Karlsruhe, Germany) and rehydrated through a descending alcohol series, followed by inactivation of the endogenous peroxidase with 0.5% hydrogen peroxide (H_2_O_2_, 30%, #9681.1, Carl Roth GmbH & Co. KG, Karlsruhe, Germany) in 85% ethanol (#K926.2, Carl Roth GmbH & Co. KG, Karlsruhe, Germany) for 30 min at room temperature. Antigen retrieval was performed by simmering in citrate buffer (pH = 6.0, #3958.1, Carl Roth GmbH & Co. KG, Karlsruhe, Germany) in the microwave (800 W) for 20 min. The primary antibodies were diluted in PBS and 1% bovine serum albumin (#0163.2, Albumin Fraktion V, Carl Roth GmbH & Co. KG, Karlsruhe, Germany) and incubated at 4 °C overnight. As negative controls, primary antibodies were replaced either by normal rabbit serum (1:3000; #R4505, Sigma–Aldrich Chemie GmbH, Tauffkirchen, Germany) or with ascites fluid from non-immunized BALB/c mice (1:1000; #BL CL8100, Cedarlane^®^, biologo, Kronshagen, Germany). Prior to incubation with the primary antibody, tissue sections were incubated with inactivated serum from the respective host species of the secondary antibody for the ABC method to prevent non-specific binding. Biotinylated goat anti-mouse (1:200; #BA-9200, Vector Laboratories, Biozol Diagnostica Vetrieb GmbH, Eching, Germany) and biotinylated goat anti-rabbit (1:200; #BA-1000, Vector Laboratories, Biozol Diagnostica Vetrieb GmbH, Eching, Germany) served as secondary antibodies, which were incubated for 45 min at room temperature. Subsequently, treatment with the avidin-biotin-peroxidase complex was conducted for 30 to 45 min at room temperature. As for the EnVision+ polymer system, tissue sections were incubated at room temperature for 30 min after overnight incubation with the primary antibodies for CD45R/B220 and SARS-CoV-2 nucleoprotein (NP). Visualization was achieved using the chromogen 3,3′-diaminobenzidine tetrahydrochloride (DAB, 0.05%, #32750-25G-F, Sigma–Aldrich Chemie GmbH, Taufkirchen, Germany) and 0.03% H_2_O_2_ for 5 min at room temperature, followed by counterstaining with Mayer’s hematoxylin (#T865.2, Carl Roth GmbH & Co. KG, Karlsruhe, Germany), dehydration and mounting with Roti^®^ Histokitt II (#T160.1, Carl Roth GmbH and Co. KG, Karlsruhe, Germany).

### 2.5. Evaluation of Results

Tissue evaluation was performed using light microscopy with the OLYMPUS BX53 (Olympus Europa SE & Co. KG, Hamburg, Germany) or on digital slide scans. The latter were obtained using the Olympus VS200 slide scanner (20× magnification for nose and lung, 40× magnification for brain and eyes, Olympus Deutschland GmbH, Hamburg, Germany) and evaluated on a 27-inch Dell UltraSharp Monitor U2722D (Dell GmbH, Frankfurt am Main, Germany).

For evaluation of the nasal tissue, RE and OE were assessed separately in sagittal sections of the nasal cavity. Using a semiquantitative scoring system (Table S2), damage to nasal mucosa was scored by evaluating severity of inflammation (0 = single inflammatory cells; 1 = 1–2 layers of inflammatory cells; 2 = 3–4 layers of inflammatory cells; 3 = 5–6 layers of inflammatory cells; 4 = ≥7 layers of inflammatory cells), distribution of inflammation (0 = no inflammation to occasional foci with few inflammatory cells but overall less than 1% of assessable tissue; 1 = 2–25% affected; 2 = 26–50% affected; 3 = 51–75% affected; 4 = >75% affected) and epithelial necrosis (0 = not observed; 1 = 2–25% affected; 2 = 26–50% affected; 3 = 51–75% affected; 4 = >75% affected) in respiratory and olfactory mucosa, respectively. In addition, hyperplastic epithelium was scored using a dichotomous scoring system (0 = no; 1 = yes) for respiratory as well as olfactory epithelium. The total respiratory and olfactory epithelium score was obtained by adding the product of distribution (0–4) and severity (0–4) of inflammation with the score for necrosis (0–4) and hyperplasia (0–1), equaling a possible range of 21 points. Finally, intraluminal exudate and vasculopathies were scored (0 = not observed to less than 1% affected; 1 = 2–25% affected; 2 = 26–50% affected; 3 = 51–75% affected; 4 = >75% affected) for the overall nasal cavity. The total nasal score results from the addition of the respiratory epithelium, the olfactory epithelium, the intraluminal exudate, and the vasculopathy score, ranging between 0 and 50 points.

Lung tissue was assessed using a previously published semiquantitative scoring system for lesions in the alveolar, vascular, and airway areas [46,47].

**Figure 1:**
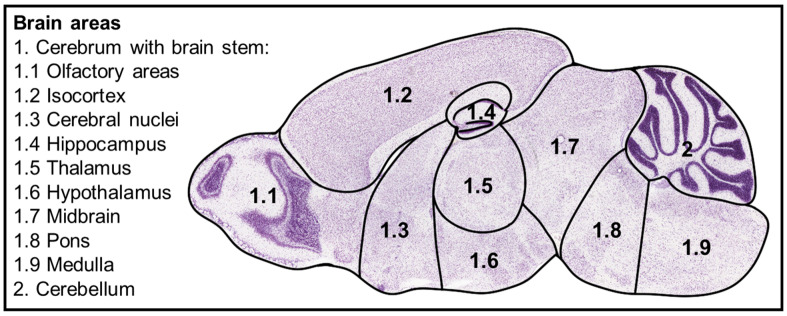
Schematic of the brain areas evaluated. The figure was adapted from the Allen Mouse Brain Atlas, mouse.brain-map.org, and atlas.brain-map.org [49]. 1.1, olfactory areas; 1.2, isocortex; 1.3, cerebral nuclei; 1.4, hippocampus; 1.5, thalamus; 1.6, hypothalamus; 1.7, midbrain; 1.8, pons; 1.9, medulla; 2, cerebellum. Nissl stain.

The brain tissue, longitudinal brain sections were divided into 10 areas such as olfactory areas (1.1), isocortex (1.2), cerebral nuclei (1.3), hippocampus (1.4), thalamus (1.5), hypothalamus (1.6), midbrain (1.7), pons (1.8), medulla oblongata (1.9), and cerebellum (2; Figure 1). “Whole brain” refers to the evaluation of the full longitudinal brain section, including all 10 areas. Brain areas were evaluated using a semiquantitative scoring system on hematoxylin and eosin-stained sections (Table S3). The severity of brain lesions was determined by the extent (0 = no inflammatory cells, 1 = single perivascular inflammatory infiltrates, 2 = 2–3 layers of perivascular infiltrates, 3 = >3 layers of perivascular infiltrates) and distribution of perivascular cell infiltration (0 = no inflammation, 1 = <50% of vessels, 2 = 50–75% of vessels, 3 = >75% of vessels). In addition, the presence or absence of vasculitis and cell death in the vascular wall and/or perivascular space (0 = no, 1 = yes) was assessed. The total vascular score results in a possible value range of 0–11 calculated by multiplying the score for the extent of perivascular inflammation (0–3) by the score for the distribution of perivascular inflammation (0–3), and adding the result to the scores for vascular lesions (0–1) and cell death in vessel wall/perivascular space (0–1). The arithmetic mean of the vascular scores from all the brain regions examined was calculated for each animal. Furthermore, the morphology of Iba-1-positive cells was assessed as previously published [48].

Globes, including retinal layers, and optic chiasms were examined separately and scored using a dichotomous scoring system (0 = no; 1 = yes) for both lesions and immunoreactivity for the respective antibodies used (Table S1).

### 2.6. Digital Analysis of Immunohistochemical Results

Quantitative and semiquantitative evaluations of digitalized tissue sections were performed using the open-source software QuPath, version 0.4.3, for the brains, eyes, and noses, for digital image analysis in pathology [50]. Stained eyes, brains, noses, and lungs (right medial lung lobe) were evaluated on longitudinal tissue sections. Individual color deconvolution was performed for all analyzed images in order to guarantee an optimal identification of the hematoxylin, eosin, and DAB signals on each slide. Retinal tissue for the evaluation of immunopositive Müller glia, as well as nasal tissue for the evaluation of immunopositive epithelial cells, were annotated manually by a veterinary pathologist.

Brain and lung tissue were automatically detected for the Iba-1 evaluation by digital thresholding. Recognized brain tissue was manually divided into 10 areas of interest (Figure 1). Nasal epithelium was manually outlined either as respiratory or olfactory epithelium. This allowed the separate digital analysis of the respiratory and olfactory epithelium.

Cell counts of CD3-immunopositive cells in the brains were obtained with a “positive cell count command” using a mean cellular DAB signal threshold. The percentage of immunopositive area was determined for SARS-CoV-2-NP and Iba-1 in the brain using a pixel classifier. In the ocular tissue, the number of immunopositive cells per area examined was obtained in the retina by manual annotation. In the nasal cavity, the percentage of positive cells was calculated using the “positive cell count command” based on a DAB channel threshold for all slides by dividing the number of positively detected cells by the total number of all detected cells in the corresponding epithelium (respiratory or olfactory epithelium). Regarding lung tissue, individual cells were subsequently identified using automated cell detection based on marker-specific nuclear thresholds. Furthermore, an object classifier based on a machine learning algorithm (Random Trees, RTrees) was trained on three representative images per staining to determine the number of immunolabeled cells by annotating ≥100 immunopositive and immunonegative cells per image. The total number of detected and immunolabeled cells per animal and per staining, as well as the corresponding percentages of immunolabeled cells, were calculated using Microsoft Excel version 2408 (Build 17928.20114; Microsoft Corp., Redmond, WA, USA) for Windows. To account for false-positive detections, the highest percentage of detected immunolabeled cells observed in the PBS group was subtracted from all other values, serving as a staining-specific correction factor. Finally, each digitally evaluated slide was manually assessed by a veterinary pathologist to verify that the automated features were executed correctly.

### 2.7. Real-Time RT-qPCR

To determine viral RNA titers in lungs and brains, the right lung lobe and the right brain hemisphere were sampled. The tissues were homogenized within 1 mL of DMEM containing antibiotics (penicillin and streptomycin, Gibco, Grand Island, NY, USA). The isolation of RNA was conducted with the KingFisher Flex and NucleoMag RNA kit following the manufacturer’s protocol. Isolated RNA from homogenates was amplified using RT-qPCR (quantitative real-time reverse transcription PCR) targeting the RNA-dependent RNA polymerase (RDRP) of SARS-CoV-2. For this, isolated RNA was incubated with the Luna^®^ Universal One-Step RT-qPCR Kit (NEB #E3005, New England Biolabs GmbH, Frankfurt am Main, Germany) in a CFX96-Touch Real-Time PCR system (Bio-Rad, Hercules, CA, USA) using the RT-qPCR program as follows. Incubation started with reverse transcription at 55 °C for 10 min, followed by denaturation at 95 °C for 1 min. In 44 cycles, samples were heated at 95 °C for 20 s for denaturation and at 56 °C for 30 s for annealing and elongation. The detection of RNA targeting RDRP of SARS-CoV-2 was conducted using the following primers: SARS-2-IP4, forward primer (5′-GGTAACTGGTATGATTTCG-3′), reverse primer (5′-CTGGTCAAGGTTAATATAGG-3′), and probe (5′-TCATACAAACCACGCCAGG-3′ [5′] FAM, [3′] BHQ-1). Measurements of relative fluorescence units (RFUs) at the time point of termination of the elongation step were conducted. A standard RNA transcript was used to correlate Ct values of the samples and calculate the quantity of viral copies per µL of total RNA.

### 2.8. Statistical Analysis

Statistical analysis was performed using SPSS for Windows^TM^ v29 (IBM^®^ SPSS^®^ Statistics, SPSS Inc., Chicago, IL, USA) using the Kruskal–Wallis test followed by the Dunn–Bonferroni procedure to investigate significant differences between the groups. Potential differences between the respiratory and olfactory epithelium within SARS-CoV-2-infected animals were investigated using Mann–Whitney U-testing. Statistical significance was accepted at a *p*-value of ≤0.05 (*). The graphs were created with GraphPad Prism version 10.2.3 (GraphPad Software, Inc., San Diego, CA, USA) for Windows™ software. Due to the homogeneity and overall low amount of background lesions among all control groups, mock-infected control animals from all endpoints (3, 6, and 7/8 dpi) were pooled. Since only one animal survived the planned necropsy at 8 dpi—the other animals died at 7 dpi or had to be euthanized because they reached the humane endpoint—both time points were combined for the statistical analysis.

## 3. Results

### 3.1. Nasal Tissue

Histologic and immunohistochemical analysis of the nasal cavity revealed transient, mild to moderate pathohistological changes in SARS-CoV-2-infected animals. Qualitative and semiquantitative analyses were performed for OE and RE, respectively.

#### 3.1.1. Histology of Nasal Tissue

##### Histology of Olfactory Epithelium

**Figure 2:**
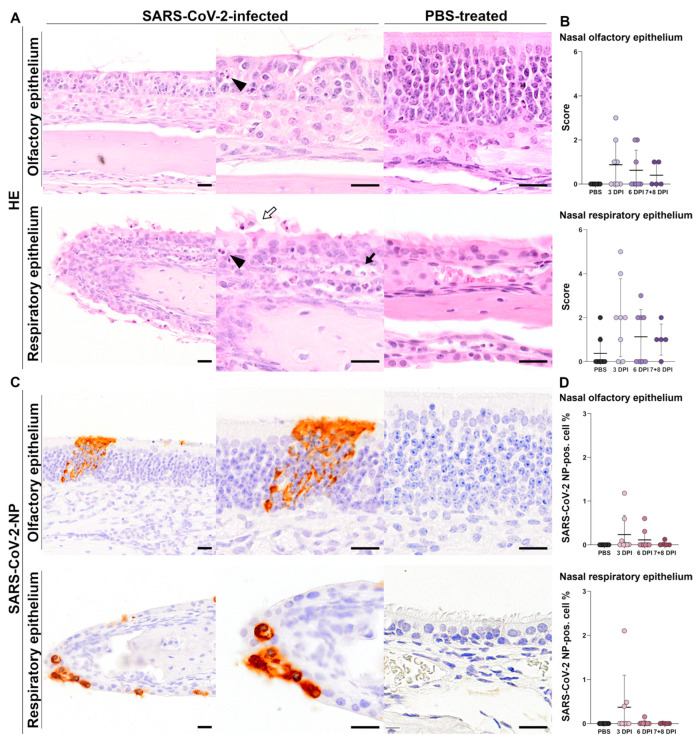
SARS-CoV-2-infected K18-hACE2 mice show mild inflammatory lesions with low amounts of epithelial SARS-CoV-2 nucleoprotein (SARS-CoV-2-NP) antigen in the nasal cavity. (**A**) Representative nasal sections of olfactory epithelium (OE) and respiratory epithelium (RE), stained with hematoxylin and eosin (HE), of SARS-CoV-2-infected animal (left, low and high magnification) and mock-infected animal (right, high magnification). OE of SARS-CoV-2-infected animal 3 days post-infection (dpi) with mild loss of epithelial architecture, fine strands of intraluminal mucus, occasional intraepithelial and nuclear fragmentation with karyopyknosis and -rrhexis (arrowhead). RE of SARS-CoV-2-infected animal 3 dpi with mild single-cell death characterized by karyopyknosis and -rrhexis (arrowhead), desquamation of ciliated cells (white arrow), and mild sub- and intraepithelial infiltration (exocytosis) of neutrophils (black arrow) compared to intact RE in a mock-infected control animal. (**B**) Semiquantitative scoring of histopathological nose lesions in the OE and RE of SARS-CoV-2- and mock-infected animals. (**C**) Representative nasal sections of OE and RE immunolabeled for SARS-CoV-2-NP of SARS-CoV-2-infected animal (left, low and high magnification) and mock-infected animal (right, high magnification). OE of SARS-CoV-2-infected animal 7/8 dpi presents low numbers of positive epithelial cells with cytoplasmic immunolabeling. SARS-CoV-2-NP was not detected in the OE of mock-infected control animals. RE of SARS-CoV-2-infected animal at 3 dpi presents low numbers of positive epithelial cells with cytoplasmic immunolabeling. SARS-CoV-2-NP was not detected in the RE of mock-infected control animals. (**D**) Quantification of SARS-CoV-2-NP immunolabeling in the total OE and RE of SARS-CoV-2 and mock-infected animals. Bars: 20 µm. The graphs show mean (solid line), individual values (dots), and standard deviation (vertical bars). Data were tested using the Kruskal–Wallis test followed by Dunn–Bonferroni post hoc testing.

Qualitative histologic analysis of the OE in SARS-CoV-2-infected groups revealed none to mild multifocal lesions, commonly located in the dorsal meatus. Lesions consisted of multifocal loss of epithelial architecture including cell loss (erosion) with low amounts of intraluminal cell debris, intraepithelial neutrophilic infiltrates (exocytosis), and single-cell detachment with cytoplasmic and nuclear condensation (apoptosis) and nuclear pyknosis or karyorrhexis and -lysis (single-cell necrosis) in basal, neuronal and sustentacular olfactory epithelial layers (Figure 2A). For better illustration of histopathologic changes induced by SARS-CoV-2 over time, nasal sections were subsequently evaluated using a semiquantitative scoring system (Table S3). Inflammatory lesions in the OE of SARS-CoV-2-infected animals were transient and most severe at 3 dpi (Figure 2B). In the OE of mock-infected control animals, no pathohistological lesions were detected (0/6).

##### Histology of Respiratory Epithelium

In the SARS-CoV-2-infected groups, 6/8 animals at 3 dpi, 4/8 animals at 6 dpi, and 4/5 animals at 7/8 dpi showed histologic lesions. At 3 dpi, qualitative histologic evaluation of the RE revealed mild, multifocal and randomly distributed neutrophilic infiltration in the lamina propria (rhinitis), often accompanied by intraepithelial neutrophilic exocytosis, multifocal loss of motile cilia and multifocal desquamation of epithelial cells (atrophy) as well as individual cells with karyopyknosis, -rrhexis and -lysis (single-cell necrosis; Figure 2A). At 6 and 7/8 dpi, SARS-CoV-2-infected animals displayed rare mild, subepithelial, mostly perivascular, lymphoplasmahistiocytic and neutrophilic infiltrates accompanied by morphologically intact respiratory epithelial cells. Semiquantitative analysis of RE revealed that inflammatory changes peaked at 3 dpi (Figure 2B). In the RE, 3/6 mock-infected control animals presented mild, multifocal, subepithelial neutrophilic and lymphohistiocytic inflammation with occasional plasmacytic rhinitis, localized in the rostral nasal respiratory epithelium.

#### 3.1.2. Immunohistochemistry of Nasal Tissue

##### SARS-CoV-2-NP Immunohistochemistry of Olfactory Epithelium

Qualitative analysis of SARS-CoV-2-NP in the nasal OE of SARS-CoV-2-infected animals sacrificed at 3 dpi showed mild, multifocal, intracytoplasmic immunolabeling for SARS-CoV-2-NP in all layers of OE cells and occasionally in luminal epithelial debris. Foci of viral SARS-CoV-2-NP antigen were inter alia frequently located in the dorsal meatus and occasionally associated with the inflammatory lesions described above in the HE analysis. At 6 dpi and 7/8 dpi, declining amounts of SARS-CoV-2-NP-positive cells were dispersed in the olfactory epithelium, often lacking an associated inflammatory lesion (Figure 2C). No viral SARS-CoV-2-NP antigen was detected in the underlying lamina propria or Bowman glands.

The amount of SARS-CoV-2-NP in the OE was quantified by calculating the percentage of all detectable positively immunolabeled epithelial cells compared to all detectable epithelial cells in the OE (Figure 2D). Overall, quantitative analysis revealed very low percentages of up to 1.18% (range 0–1.18%) of infected epithelial cells in the OE, with its peak at 3 dpi. At this time point, although not significant, a higher percentage of SARS-CoV-2-NP-positive cells was detectable in the OE of SARS-CoV-2-infected animals compared to mock-infected control animals (*p* = 0.137). Thereafter, the amount of SARS-CoV-2-NP viral antigen gradually decreased over 6 dpi and 7/8 dpi, with only single immunolabeled cells (max. 0.13% SARS-CoV-2-NP-positive OE cells per all detected OE cells) present in individual animals at 7/8 dpi. No viral antigen was detectable in the OE of PBS-treated control animals.

##### SARS-CoV-2-NP Immunohistochemistry of Respiratory Epithelium

Qualitative analysis of SARS-CoV-2-NP in the RE of SARS-CoV-2-infected animals sacrificed at 3 dpi presented low amounts of multifocal, randomly distributed SARS-CoV-2-NP antigen in multiciliated epithelial cells, occasionally associated with motile cilia, in non-ciliated epithelial cells, and occasionally in desquamated epithelial cells (Figure 2C). Foci of SARS-CoV-2-NP immunolabeling were occasionally, but not always, associated with inflammatory lesions described above in the HE analysis. At 6 dpi, multifocal single SARS-CoV-2-NP-positive cells were detected, often lacking an associated inflammatory lesion. No viral SARS-CoV-2-NP antigen was detected by qualitative analysis in the RE at 7/8 dpi. Furthermore, no SARS-CoV-2-NP-positive cells were detected in the nasal-associated lymphoid tissue (NALT) at any time point.

Quantitative analysis of SARS-CoV-2-NP in the RE was performed as described for the OE (Figure 2D). The amount of SARS-CoV-2-NP-positive respiratory epithelial cells was overall low (range 0–2.101%) at all time points. At 3 dpi, 3/8 SARS-CoV-2-infected animals showed a mean percentage of 0.372% of SARS-CoV-2-NP-positive epithelial cells in the RE. At 6 dpi, only one animal displayed single positive respiratory epithelial cells (0.21% SARS-CoV-2-NP-positive cells per all detected cells), whereas the percentage of SARS-CoV-2-NP-immunopositive cells detectable by digital image analysis was below 0.01% at 7/8 dpi. No viral antigen was detectable in the RE of PBS-treated control animals.

### 3.2. Lung Tissue

#### 3.2.1. Histology of Lung Tissue

**Figure 3:**
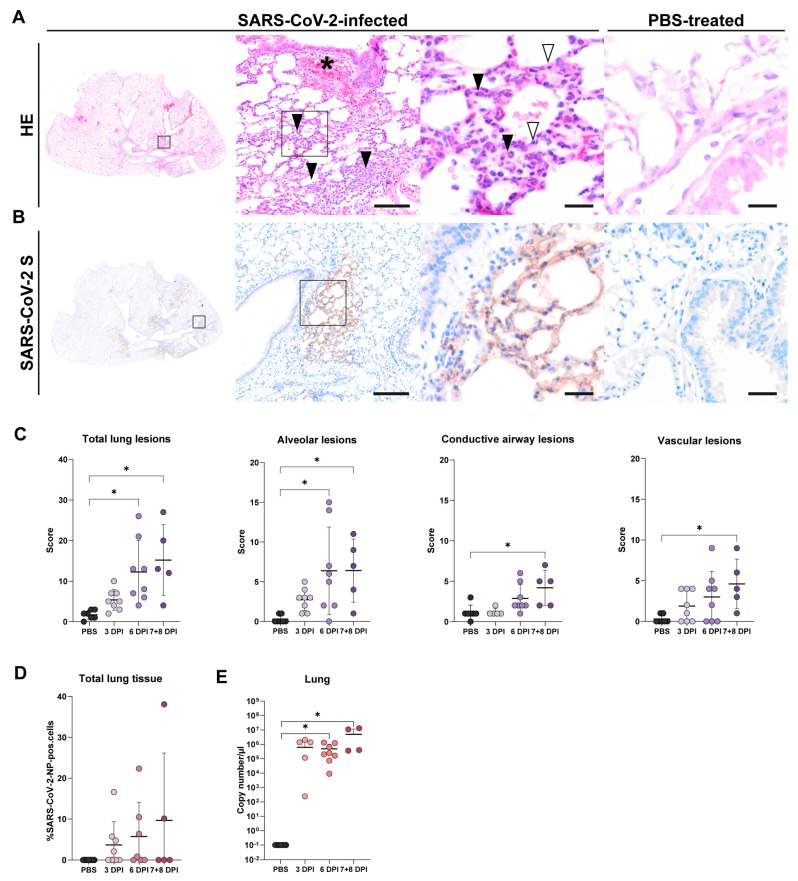
Progressing pneumonia and SARS-CoV-2 nucleoprotein (SARS-CoV-2-NP) antigen expression in lungs of K18-hACE2 mice during the initial 8 days of infection with SARS-CoV-2. (**A**) Representative images of lung lesions in infected animals compared to PBS-treated control tissue. Hematoxylin and eosin (HE) stained overview and higher magnification images show multifocal areas of septal and interalveolar immune cell infiltration (black arrowheads) as well as perivascular hemorrhage (asterisk). Higher magnification reveals multifocal single-cell necrosis within alveolar septae (white arrowheads, SARS-CoV-2-infected animal at 6 dpi). (**B**) Representative images of pulmonary SARS-CoV-2-NP in infected animals compared to PBS-treated controls. SARS-CoV-2-NP antigen was expressed multifocally throughout the alveolar tissue (brown signal). High magnification image shows SARS-CoV-2 S antigen expression in type I and II pneumocytes at 6 days post-infection (dpi). Rectangles indicate magnified areas in subsequent images. Bars = 100 and 20 µm, respectively. (**C**) Semiquantitative scoring of histopathological lung lesions. (**D**) Quantitative analysis of SARS-CoV-2-S immunolabeling in the total lung tissue of infected mice compared to mock-infected mice. (**E**) RT-qPCR targeting RNA-dependent RNA polymerase (RDRP) of SARS-CoV-2 in lung tissue of SARS-CoV-2-infected mice compared to mock-infected mice. The graphs show mean (solid line), individual values (dots), and standard deviation (vertical bars). Data were tested using the Kruskal–Wallis test followed by Dunn–Bonferroni post hoc testing. Statistical significance was accepted at a *p*-value of ≤0.05 (*).

Lung lesions in SARS-CoV-2-infected animals were mainly observed in the alveolar compartments and showed large variations at certain time points. At 3 dpi, multifocal small areas with minimal to mild septal and luminal infiltrates (2–3 cells thick), consisting mainly of neutrophils and macrophages, were observed. At 6 dpi, histologic changes presented a multifocal to coalescent distribution and affected between 51 to 75% of the tissue. Alveolar changes were characterized by moderate to marked septal and luminal infiltration by neutrophils, macrophages, as well as lymphocytes, often completely obscuring alveolar architecture, with single-cell necrosis, fibrin extravasation, and alveolar hemorrhage (4/9) or minimal to mild changes as described above (4/9; Figure 3A). No alveolar lesions were observed in one animal. At 7/8 dpi, alveolar lesions were mainly minimal to mild, with only 1/6 animals showing moderate lesions. Lesions within the conductive airways and vascular compartment were minimal to mild at all time points and affected between 2 and 25% of the tissue. Most infected animals showed minimal to mild bronchiolitis or perivascular infiltration of up to two layers of macrophages and neutrophils, comparable to inflammatory changes seen in mock-infected animals. More pronounced lesions were only observed in one animal at 6 dpi, which showed between 3 and 5 cell layers of perivascular cuffs around most vascular cross sections. Non-inflammatory lesions in these compartments included mild to moderate hyperplasia of bronchiolar epithelial cells (18/22) and pronounced perivascular hemorrhage (4/23; Figure 3A) or edema (1/23). Histopathological assessment of PBS-treated control animals revealed minimal infiltration of the alveolar (2/7), airway (3/7), and vascular (2/7) compartment by a few inflammatory cells consisting mainly of neutrophilic granulocytes, macrophages, or lymphocytes.

Despite an overall high variance in total lung histological scores at 6 and 7/8 dpi, significant differences were observed at both time points compared to mock-infected animals (*p* = 0.001, *p* = 0.002, respectively). As indicated, changes were most pronounced in the alveolar compartment, with significant differences to mock-infected animals at 6 dpi (*p* = 0.007) and 7/8 dpi (*p* = 0.008). Lesions in the conductive airway and vascular compartment were only statistically significant at 7/8 dpi (*p* = 0.018, *p* = 0.027, respectively; Figure 3B).

#### 3.2.2. SARS-CoV-2-NP Immunohistochemistry of Lung Tissue

SARS-CoV-2-NP antigen was observed multifocally in the alveolar compartment of infected mice at all time points examined, while only low levels of signal were observed in the conductive airway or vascular compartment (Figure 3A). As illustrated, expression was mainly present in type I and II pneumocytes, and occasionally in infiltrating macrophages. After a correction for background detection in mock-infected animals, immunoreactivity of <1% of detected cells was observed in 4/9 animals at 3 dpi and 2/9 animals at 6 dpi. Expression of SARS-CoV-2-NP in more than 1% of all detected cells was observed in 3/9 animals at 3 dpi (4.70–16.58%), 3/9 animals at 6 dpi (6.34–22.35%), and 2/6 animals at 7/8 dpi (10.10 and 38.09%; Figure 3D). No viral antigen was detectable in the lung of PBS-treated control animals (0/7).

#### 3.2.3. RT-qPCR of Lung Tissue

RT-qPCR analysis targeting the RDRP of SARS-CoV-2 revealed high titers in the lungs. RNA levels were similar at 3 and 6 dpi, with increased titers at 7/8 dpi (Figure 3E). Significant differences were reached in infected animals at 6 dpi (*p* = 0.022) and 7/8 dpi (*p* = 0.016) when compared to the mock-infected animals.

### 3.3. Brain Tissue

#### 3.3.1. Histology of Brain Tissue

**Figure 4:**
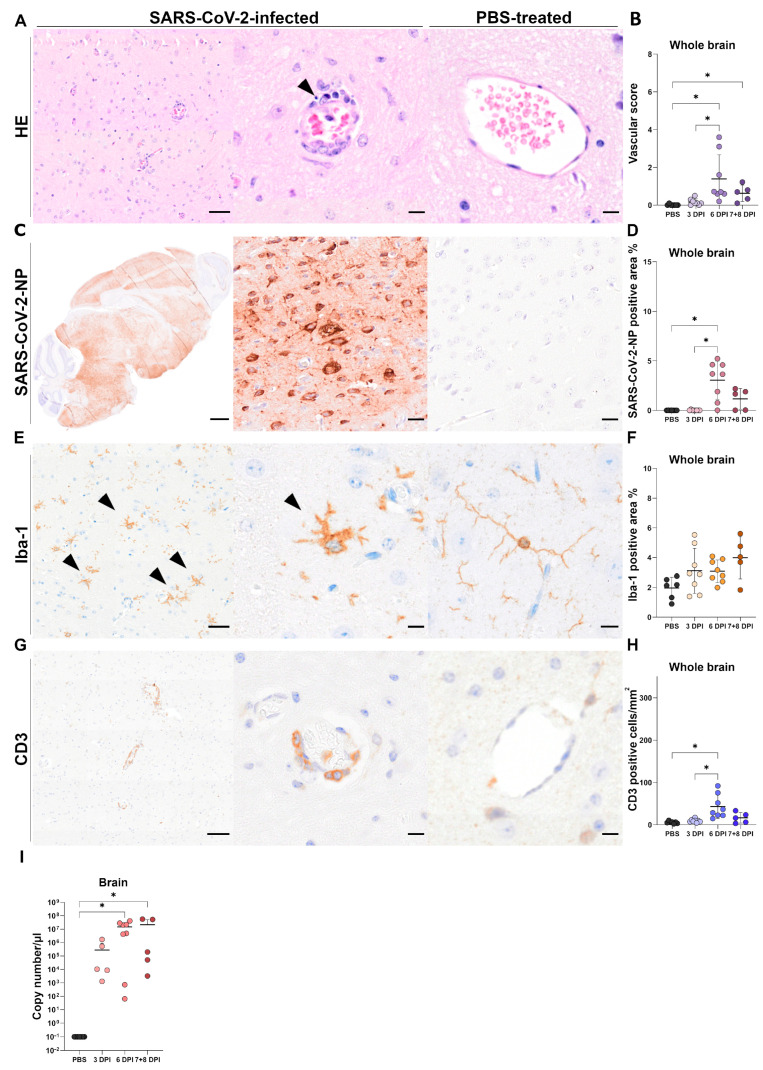
SARS-CoV-2-infected K18-hACE2 mice show lymphohistiocytic meningoencephalitis with microgliosis and diffuse intraneuronal SARS-CoV-2 nucleoprotein (SARS-CoV-2-NP) antigen. (**A**) Representative images of the thalamus stained with hematoxylin and eosin (HE) showing predominantly perivascular mononuclear infiltration with the presence of pyknotic nucleus (arrowhead) in infected animal 6 days post-infection (dpi; left, low and high magnification), and lack of inflammatory infiltrates in the mock-infected animal (right, high magnification). (**B**) Semiquantitative scoring of histopathological lesions in whole brains (cerebrum and cerebellum), with peak of inflammatory changes occurring in the infected animals at 6 dpi. Histological lesions, although less pronounced, were also present at 7/8 dpi. (**C**) Representative brain sections immunolabeled for SARS-CoV-2-NP with diffuse, neuronal viral antigen spread throughout the cerebrum and brain stem, but not cerebellum, in the infected animal at 6 dpi (left, low and high magnification), and no positive staining in the thalamus of the mock-infected animal (right, high magnification). (**D**) Quantification of SARS-CoV-2-NP-positive area (%) in the whole brain (cerebrum and cerebellum). (**E**) Representative thalamus sections immunolabeled for Iba-1 presenting hypertrophied microglial morphology characterized by shortened, thickened processes in the infected animal at 6 dpi (left, low and high magnification) in contrast to ramified microglia with long, thin processes in the mock-infected animal (right, high magnification). (**F**) Quantification of Iba-1-positive area (%). (**G**) Representative thalamus section immunolabeled for CD3, demonstrating the presence of numerous T cells in the perivascular infiltrates in the infected animal at 6 dpi (left, low and high magnification), and absence of perivascular T cell infiltrates in the mock-infected animal (right, high magnification). (**H**) Quantification of CD3-positive cells in the whole brain. (**I**) RT-qPCR targeting RNA-dependent RNA polymerase (RDRP) of SARS-CoV-2 in brain tissue of SARS-CoV-2-infected mice compared to mock-infected mice. Bars: overview = 1 mm; low magnification = 50 µm; high magnification = 10 µm. The graphs show mean (solid line), individual values (dots), and standard deviation (vertical bars). Data were tested using the Kruskal–Wallis test followed by Dunn–Bonferroni post hoc testing. Statistical significance was accepted at a *p*-value of ≤0.05 (*).

**Figure 5:**
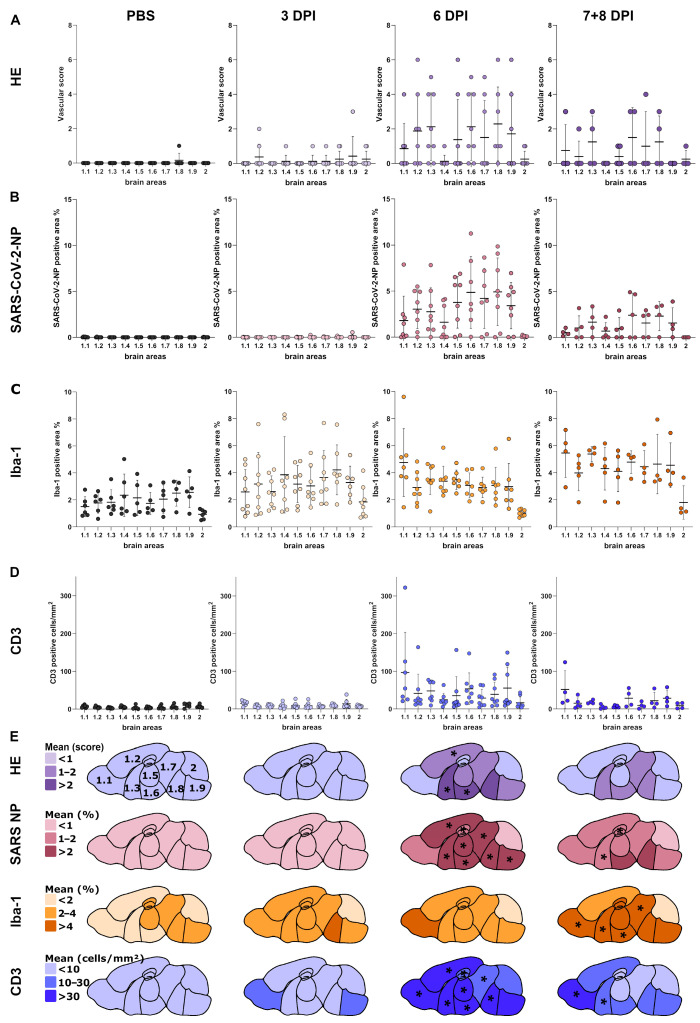
Histological lesions, viral antigen, and T cell infiltration in SARS-CoV-2-infected K18-hACE2 mice were most prominent at 6 dpi, while the highest Iba-1 immunoreactivity was observed at 7/8 dpi. (**A**–**D**) Quantification of histopathology on hematoxylin and eosin-stained brains (**A**), semiquantitative score), SARS-CoV-2-NP-positive area (**B**), Iba-1-positive area (**C**), and CD3-positive T cells (**D**), in separate brain regions (see also Figure 1). (**E**) Schematic representation of quantitative data. The color scheme is used to visualize quantitative differences. Asterisks indicate significant differences between infected animals sacrificed at 3, 6, or 7/8 dpi and mock-infected controls in the respective brain region. Data were tested using the Kruskal–Wallis test followed by Dunn–Bonferroni post hoc testing. Statistical significance was accepted at a *p*-value of ≤0.05 (*). The graphs show mean (solid line), individual values (dots), and standard deviation (vertical bars).

Animals infected with SARS-CoV-2 showed mild to moderate, lymphohistiocytic, predominantly perivascular meningoencephalitis throughout the cerebrum and brain stem (Figure 4A), but none to minimal inflammation in the cerebellum. Minimal inflammatory changes were observed in some of the infected animals as early as 3 dpi (5/8), whereas at 6 dpi, lesions were most prominent with moderate perivascular infiltrates (up to three layers), and 75% of the blood vessels were affected. Additionally, the semiquantitative vascular score for the whole brain was statistically significant in infected animals at 6 dpi in comparison to the mock-infected animals (*p* < 0.001), as well as infected animals at 3 dpi (*p* = 0.042; Figure 4B). At 6 dpi, histological changes were especially pronounced and significantly higher compared to mock-infected animals in the multiple brain regions, such as the isocortex (*p* = 0.042), cerebral nuclei (*p* = 0.009), and hypothalamus (*p* = 0.026), reaching the statistical significance level (Figure 5A,E). Inflammatory changes at 7/8 dpi (4/5) regarding the whole brain were less prominent than at 6 dpi, but significant in comparison to the mock-infected group (*p* = 0.016). In addition, 4/8 of the animals at 6 dpi and 3/5 of the animals at 7/8 dpi had variable numbers of swollen and severely vacuolated neurons, primarily located in the isocortex and to a lesser extent in the hippocampus and mitral layer of the olfactory bulb (Figure S1). Histopathological assessment of PBS-treated control animals revealed minimal infiltration of the meningeal (1/6), parenchymal (2/6), and perivascular (1/6) compartment by single lymphocytes.

#### 3.3.2. Immunohistochemistry of Brain Tissue

##### SARS-CoV-2-NP Immunohistochemistry

At all time points, viral antigen was only present in the neuronal cytoplasm, and the positive cells were predominantly located in the cerebrum and brain stem of SARS-CoV-2-infected animals (Figure 4C). In the cerebellum, SARS-CoV-2-NP-positive cells were mainly observed in the nuclei and rarely in the Purkinje cell layer. At 3 dpi, only 2/8 animals showed focal to multifocal, patchy areas with SARS-CoV-2-NP-positive neurons in multiple brain regions, including cerebral nuclei, hypothalamus, midbrain, pons, medulla oblongata, and single cells in the olfactory bulb. However, at 6 dpi, viral antigen was present in the brain of 7/8 animals, and SARS-CoV-2-NP-positive area measurement was significantly higher in comparison to mock-infected animals (*p* = 0.011) and to the infected animals at 3 dpi (*p* = 0.001; Figure 4D). Viral antigen was distributed either multifocally (1/8) or diffusely (6/8) throughout the cerebrum. Interestingly, in the one animal showing a multifocal distribution pattern of viral antigen, positive cells were observed mainly in the basal regions of the brain as well as the mitral cell layer of the olfactory bulb, hippocampus, and isocortex (laminar pattern). Quantitative evaluation of the staining revealed significant differences in the percentage of the SARS-CoV-2-NP-positive area in the following brain regions: isocortex, *p* = 0.004; cerebral nuclei, *p* < 0.001; hippocampus, *p* = 0.002; thalamus, *p* < 0.001; hypothalamus, *p* = 0.002; midbrain, *p* = 0.005; pons, *p* = 0.003; medulla, *p* = 0.001;) of infected mice in comparison to the mock-infected group. At 7/8 dpi, 3/5 of the infected animals showed multifocal to coalescing (2/5) or diffuse (1/5) spread of viral nucleocapsid protein in the cerebrum, but only the cerebral nuclei (*p* = 0.013) and hippocampus (*p* = 0.038) reached the statistical significance level (Figure 5B,E). No viral antigen was detected in mock-infected control animals (0/6).

##### Iba-1 Immunohistochemistry

At 3 dpi, SARS-CoV-2-infected animals, like mock-infected animals, presented microglia characterized by long, thin processes and small, round somas. In contrast, microglia in SARS-CoV-2-infected mice showed altered morphology and more frequent vacuolization of the cytoplasm at 6 dpi and 7/8 dpi. Activated spiky microglia, characterized by shorter, thicker processes and enlarged somas (Figure 4E), were frequently observed, as well as, though less prominent, amoeboid and occasional hyper-ramified microglia. In most of the infected animals at 6 and 7/8 dpi, the change in microglial morphology was diffuse, in a few mice being multifocal or multifocal to coalescing. The increased whole brain Iba-1-positive area at 3, 6, and 7/8 dpi did not reach statistical significance when compared to the mock-infected animals. However, the most prominent increase was observed at 7/8 dpi (*p* = 0.059; Figure 4F). In addition, more detailed quantification of the staining revealed significant differences compared to the mock-infected animals in the olfactory areas (*p* = 0.022), cerebral nuclei (*p* = 0.004), hypothalamus (*p* = 0.006), and midbrain (*p* = 0.039; Figure 5C,E).

##### CD3 Immunohistochemistry

CD3 staining highlighted a predominantly perivascular and, to a lesser extent, parenchymal infiltration of T cells in SARS-CoV-2-infected animals (Figure 4G). At 3 dpi, only occasional, single-cell infiltrates were observed, similar to the mock-infected control animals. At 6 dpi, the infiltrates were most prominent, and the number of CD3-positive cells detected in the whole brain was significantly higher than in the mock-infected animals (*p* < 0.001) and the infected animals at 3 dpi (*p* = 0.045; Figure 4H). In addition, a more detailed analysis revealed significant increase in the number of CD3-positive cells in the olfactory areas (*p* < 0.001), isocortex (*p* = 0.001), cerebral nuclei (*p* < 0.001), hippocampus (*p* = 0.038), thalamus (*p* = 0.003), hypothalamus (*p* = 0.001), midbrain (*p* = 0.018) and pons (*p* = 0.010). At 7/8 dpi, statistical significance was present only in the olfactory areas (*p* = 0.025) and cerebral nuclei (*p* = 0.024; Figure 5D,E).

##### CD45R and Myeloperoxidase Immunohistochemistry

Only single B lymphocytes and neutrophilic granulocytes were detected in the brains of infected and mock-infected animals.

##### Beta-Amyloid Precursor Protein, Kinesin, and Neurofilament Immunohistochemistry

**Figure 6:**
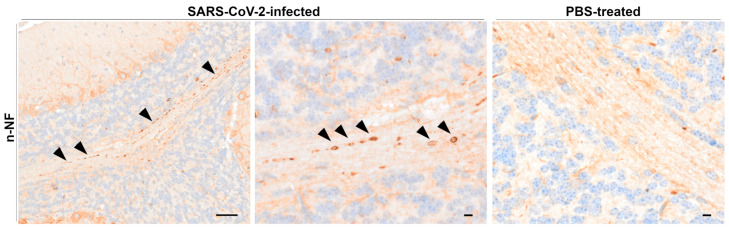
Axonal damage in SARS-CoV-2-infected K18-hACE2 mice. Multifocal round to oval accumulations of non-phosphorylated neurofilament (nNF; arrowheads) were observed in the cerebellar white matter of SARS-CoV-2-infected K18-hACE2 mice at 6 dpi (left, low and high magnification), but not in the control animals (right, high magnification). Bar = 50 µm.

Cerebellar white matter of some of the infected animals at 6 dpi (4/8) and 7/8 dpi (3/5) showed multifocal, round to oval accumulations of non-phosphorylated neurofilament (nNF), occasionally aligned into beaded-like structures (Figure 6). No such finding was observed in infected animals sacrificed at 3 dpi or mock-infected control animals.

Immunohistochemistry for beta-amyloid precursor protein (β-APP), motor protein kinesin, and phosphorylated neurofilament (pNF) did not show any aberrations indicative of axonal damage.

#### 3.3.3. RT-qPCR of Brain Tissue

RT-qPCR analysis targeting the RDRP of SARS-CoV-2 revealed high titers in the brain that increased from 3 to 6 dpi, with the highest amounts of RNA found at 7/8 dpi (Figure 4I). Statistical significance was observed at 6 dpi (*p* = 0.002) and 7/8 dpi (*p* = 0.008) when compared to the mock-infected animals.

### 3.4. Globes and Optic Chiasms

#### 3.4.1. Histology of Globes and Optic Chiasms

Examination of globes, including all retinal layers, and optic chiasms yielded no pathomorphological alterations on the specimens stained with hematoxylin and eosin, cresyl fast violet, and alcian blue, for neither vascular changes nor leukocyte infiltration, degeneration, or necrosis at any evaluated time point.

#### 3.4.2. Immunohistochemistry of Globes

##### SARS-CoV-2-NP Immunohistochemistry

**Figure 7:**
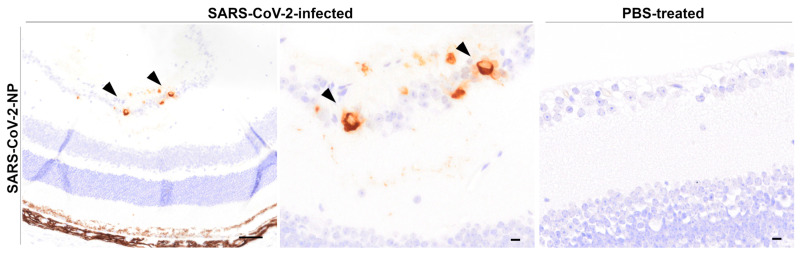
Retinal infection in SARS-CoV-2-infected K18-hACE2 mice. SARS-CoV-2-NP immunohistochemistry of retinas from infected K18-hACE2 mice reveals the presence of multiple neurons with intracytoplasmic SARS-CoV-2-NP antigen (arrowheads) at 6 dpi (left, low and high magnification), but not in the control animals (right, high magnification). Bar = 50 µm.

At 6 dpi, viral-antigen-positive neurons (ganglion cell layer, inner nuclear cell layer) were observed in the retinas of 4/7 infected animals (Figure 7). At 7/8 dpi, positive neurons (ganglion cell layer) were seen in 2/5 animals. Viral antigen was present most frequently in retinal neurons of the ganglion cell layer (6/6) and additionally in the inner nuclear cell layer in one of the animals (1/6). Animals sacrificed at 3 dpi showed no SARS-CoV-2-NP-positive cells in the globes, as did the mock-infected control animals. No positive immunostaining was detected in the chiasm of the infected animals at 3 dpi (1/1), at 7/8 dpi (1/1), or in any of the mock-infected controls (3/3).

##### Iba-1, CD3, Glial Fibrillary Acidic Protein (GFAP) and Glutamine Synthetase (GS), β-Tubulin, Neurofilaments, and Caspase 3 Immunohistochemistry

Immunohistological staining for Iba-1 revealed no morphologic or quantitative changes in the retinal microglia. However, the microglia in all examined optic chiasms from infected animals at 6 dpi (3/3) and 7/8 dpi (1/1) showed shortened and thickened processes in comparison to the mock-infected animals (3/3).

Immunohistochemistry for GFAP and GS revealed no significant changes in the Müller glia of the retina in terms of increased immunolabeling, nor were morphologic or quantitative changes in astrocytes in optic chiasms detected.

Immunohistochemistry for nNF, pNF, and β-tubulin did not result in any aberrations indicative of axonal damage.

The number of caspase 3-positive cells was minimal and therefore not quantified.

## 4. Discussion

### 4.1. Olfactory Epithelium Shows Prolonged SARS-CoV-2 Infection Compared to Respiratory Epithelium in K18-hACE2 Mice

K18-hACE2 mice are well recognized as a suitable model for studying upper respiratory tract pathogenesis in the context of SARS-CoV-2 [12,16,18], even though the model fails to simulate severe and lethal cases of COVID-19, including its neuroinvasive potential without full neurodissemination [12,21,51]. Similar to COVID-19 in humans, K18-hACE2 mice show a transient and mild inflammation in the nasal cavity upon intranasal SARS-CoV-2 inoculation [12,16,18]. However, current literature mainly focuses on qualitative and descriptive histopathological analysis in the nasal cavity. Therefore, the present study aimed to gain further insight on the spatiotemporal development of ancestral SARS-CoV-2 infection in K18-hACE2 mice by examining the upper respiratory tract mucosa—discriminated into respiratory and olfactory mucosa—for pathohistological alterations alongside a quantitative analysis of intranasal viral antigen at different time points post-infection (3, 6, 7/8 dpi). In line with previous studies [18], both epithelia demonstrated mild viral infection consistent with observed inflammation patterns. This suggests that SARS-CoV-2 induces only mild inflammatory responses in the nasal cavity, irrespective of OE or RE. Interestingly, the OE showed clusters of infected cells, with SARS-CoV-2 antigen persisting until 7/8 dpi, while the RE showed a scattered distribution, with viral antigen levels falling below 0.01% at this time point. In agreement with Carossino et al. (2022), showing an olfactory tropism for SARS-CoV-2 with a presumed delayed histiocytic clearance [12], a prolonged presence of viral antigen in the OE at 7/8 dpi was confirmed by spatiotemporal quantification of viral antigen in the OE and RE, respectively. Similar findings of olfactory tropism have been described for ancestral SARS-CoV-2 strains in human nasal explants as well as Syrian golden hamsters (*Mesocricetus auratus*), wherein an impaired phagocytic function of macrophages with associated delayed SARS-CoV-2 clearance in the OE was discussed [52].

Consistent with other studies, the lack of inflammatory or reactive changes detectable in HE staining, together with the complete absence of viral antigen at all time points investigated, suggests a quiescent local immune response of the NALT [53]. However, despite the fact that it was not always present for histological analysis, it might simply be too early for germinal center reaction, which could be expected to develop later than a week post-infection.

Although the neurotropism and neuroinvasion of SARS-CoV-2 in COVID-19 patients remains the subject of ongoing debate, it is the current scientific consensus that SARS-CoV-2 does not primarily target olfactory sensory neurons to enter the human CNS [21,22,24,54,55,56,57,58,59]. Similarly, the olfactory nerve route as a pathway to the CNS has been questioned in K18-hACE2-mice [60]. In K18-hACE2-mice, SARS-CoV-2 tends to infect olfactory sustentacular cells (SUS) along with Bowman’s gland cells in the nasal cavity by binding to ACE2 and TMPRSS2 proteins [21,60]. ACE2 receptors are not only abundant but also overexpressed in the nasal cavity, which represents a potential key feature for neuroinvasion in this animal model [12]. The present results are consistent with reports of targeted infection of SUS, although viral antigen was also observed in basal and neuronal olfactory epithelial cells, while it was absent in the Bowman’s gland. Nevertheless, as the virus allegedly targets SUS, it damages the supporting structure of the olfactory system, leading to olfactory dysfunction [60]. In doing so, cell death mechanisms along with an interferon-dependent immune response likely limit the virus spread to the CNS [60]. The present study did not detect viral antigen in the lamina propria or Bowman’s gland of the OE nor the olfactory nerve fibers by immunohistochemistry, suggesting an alternative pathway to the CNS than the presumed olfactory nerve route [22,60]. Further supporting this is the limited presence of SARS-CoV-2 in the olfactory bulb [25,26,60], especially at early time points post-infection, as observed in this study, although the virus replicates in the OE after intranasal inoculation. Alternative pathways of neuroinvasion to the olfactory route include the hematogenous, gustatory, trigeminal, and vagus pathways [20,21,22,23,24,25].

### 4.2. Aveolar-Restricted SARS-CoV-2 Pneumonia in K18-hACE2 Mice

The K18-hACE2 mice have previously been shown to develop severe lung lesions following intranasal infection with SARS-CoV-2, including acute pneumonia, alveolar collapse, interstitial as well as perivascular and vascular immune cell infiltration, alveolar septal thickening, and proteinaceous debris in alveolar spaces [12,14,61,62,63,64]. While lesions in humans and other species develop first in the conductive airways and subsequently spread to the alveolar lung compartment, little to no bronchiolar changes are observed in acute infections of K18-hACE2 mice [12,61,65,66,67,68,69,70,71]. This lesion pattern suggests that the main target cells in this model are alveolar pneumocytes, in contrast to bronchiolar and then alveolar epithelia in humans and other species, such as Syrian golden hamsters and Roborovsky hamsters [65,68,71,72,73,74]. However, studies at earlier time points post-infection are warranted to further investigate the potential differences in cell tropism in K18-hACE2 mice.

While most lesions and a viral antigen expression in type I and II pneumocytes were observed in the majority of the mice examined, both the semiquantitative assessment of lesion distribution and severity as well as the quantitative evaluation of viral antigen expression revealed a high variance of pulmonary disease severity despite the overall lethal outcome in the present study. This finding was further reflected in the comparative analysis of immunohistochemistry and RT-qPCR. While a general correlation was evident, with samples showing strong immunohistochemical staining often having high viral RNA loads, some discrepancies were noted. Several lung samples exhibited detectable viral RNA despite low or absent antigen staining. These differences likely result from varying assay sensitivity and tissue coverage.

This underlines the hypothesis that the lethality of SARS-CoV-2 infection in this model is more likely related to rapid and widespread neuroinvasion rather than to pulmonary changes as observed in humans and Roborovsky hamsters [12,14,62,71,75,76,77]. In accordance with previous studies, both the observed distribution of lung lesions and the relatively low expression of viral antigen, restricted to the alveolar compartment, suggest an extrapulmonary or nervous route of neuroinvasion, as no evidence of vascular or endothelial infection was shown [12,19,26].

### 4.3. Mononuclear Meningoencephalitis and Microgliosis Represent Common Features of SARS-CoV-2 Infection

Variable neurological symptoms such as anosmia, dysgeusia, dizziness, headache, as well as impaired consciousness and stroke have been linked to the SARS-CoV-2 infection in human patients [78,79]. However, as in hamsters, neuroinvasion is less pronounced in humans, but has been verified by detection of SARS-CoV-2 RNA with RT-qPCR [23,24,59,80,81] and immunohistochemical staining for viral proteins of isolated parenchymal cells [23], cortical neurons [82], and vascular endothelial cells [24,82] within the brain. In contrast, neuroinvasion of SARS-CoV-2 with broad distribution of viral antigen is a regular finding in K18-hACE2 mice [12,14,16,19,25,26,82]. In accordance with recent reports, the present study shows that K18-hACE2 mice manifest lymphohistiocytic meningitis with extensive viral spread into the CNS.

As in previous studies [15,25,26], immunohistochemical analysis revealed a progressive increase in the number and distribution of SARS-CoV-2-NP-positive neurons. These findings are in agreement with the RT-qPCR analysis of viral RNA concentration in the brain. The appearance of SARS-CoV-2-NP protein in neurons remains slightly behind the detection of viral RNA by qPCR, likely reflecting the time required for translation and accumulation of viral proteins to detectable levels. In addition, multifocal neuronal vacuolation in the cerebral cortex, hippocampus, and olfactory bulb was present in some infected animals at 6 and 7/8 dpi. Interestingly, in the few infected mice with multifocal distribution of viral-antigen-positive neurons, the immunoreactivity correlated partly with the location of vacuolated neurons. Similar lesions have been briefly described in other studies [12,16,25]. Using electron microscopy, Carossino et al. (2022) have shown that vacuolated neurons contain numerous intracytoplasmic virus particles and various replication-associated membranous structures [12]. These findings suggest that cytoplasmic vacuolation may be a sequel to the presence of viral proteins in the cell.

The most frequently reported CNS lesions in human COVID-19 patients include predominant lymphocytic inflammation, microgliosis with microglial nodules, acute hypoxic-ischemic changes, and astrogliosis [79]. Demyelination, cerebral hemorrhage, and acute neuronal loss are reported less frequently [23,79,80,83]. Similarly, next to the broad distribution of viral antigen, K18-hACE2 mice show mild lymphohistiocytic meningoencephalitis upon SARS-CoV-2 infection [12,14,16,19,25,26,82]. The inflammatory reaction and viral spread peak at 6 and 7/8 dpi, coinciding with morbidity or mortality [12,14,16,19,25,26]. Likewise, present results show that cerebral lymphohistiocytic infiltration and spread of viral protein in the brain of SARS-CoV-2-infected K18-hACE2 mice are most prominent at 6 dpi.

Microgliosis, as observed previously [12,14,17,26], was mostly mild but diffuse (cerebrum and brain stem) in SARS-CoV-2-infected mice in this study, with the highest value at 7/8 dpi. Interestingly, animals with multifocal distribution of viral antigen also showed multifocal microgliosis, though not always associated with SARS-CoV-2-positive neurons. The activation of microglia in the infected CNS can be stimulated by cytokine signaling, damaged neuron-derived nucleotides, or direct recognition of viral antigen by microglial pattern-recognition receptors [84,85]. Importantly, in K18-hACE2 mice, the Iba-1-positive area increased as early as 3 dpi, with no clear morphological changes. This suggests an initial priming of microglia triggered by systemic inflammation rather than direct viral invasion. Indeed, a pronounced peripheral cytokine-chemokine storm is reported for K18-hACE2 mice by 2–4 dpi [13,16,19,86,87,88], consistent with subtle microglial activation as observed in this study. Although SARS-CoV-2-NP-positive neurons are minimal at 3 dpi, viral RNA is detectable in some mice by RT-qPCR, indicating low-level neurotropism below the immunohistochemical threshold.

At 5–6 dpi, the viral antigen was widely distributed throughout the brain but not the cerebellum, concurrent with a robust infiltration of T cells, followed by overt morphology change in microglia by 7/8 dpi, marking a transition from innate priming to active neuroinflammation [19,86,89,90]. Thus, the temporal pattern in K18-hACE2 mice follows an early systemic inflammation and low-level neurotropic signaling that primes microglia, followed by later, direct viral invasion that triggers morphological activation and engagement of adaptive immunity. Transition of microglia toward a proinflammatory phenotype may subsequently contribute to the exacerbation of neurodegenerative processes characteristic of chronic neurodegenerative diseases [86,91,92,93].

However, the multifocal occurrence of activated microglia in close proximity to viral antigen indicates a direct microglial response to infected cells and antigen, rather than a generalized activation as seen in response to peripheral infection [94]. In contrast to previous reports [12,15,16,17,19,25], animals from the present study did not show any evidence of thrombi or neuronal cell death, which is consistent with the report by Seehusen, Clark et al. (2022) [26].

In line with previous reports [12,26,82], limited involvement of the cerebellum was observed. The reason for this phenomenon remains obscure, but it does not appear to be related to the expression and distribution of the hACE2 receptor in this mouse model. In K18-hACE2 mice, the virus appears to spread independently of ACE2 expression, as it infects cells that do not exhibit ACE2 and does not necessarily target all ACE2-expressing cells [12,26].

In SARS-CoV-2-infected mice, brain-specific hACE2 expression has been shown to be associated with lethal outcomes, with intraventricular infection leading to death by 6 dpi [82]. In contrast, intranasally infected mice with lung-specific hACE2 expression showed no weight loss or mortality [82]. Furthermore, neurological signs and mortality in these mice correlate with peak viral titers in the brain rather than the lungs [19], suggesting a direct virus-associated neuronal dysfunction, supported by observations of severe neuroinvasion and vacuolated, virus-positive neurons [12,16,25]. These findings underline the importance of CNS involvement in SARS-CoV-2 infection of this animal model. However, the exact mechanism behind these neurological manifestations remains unclear.

In humans, the generally low number of SARS-CoV-2-positive cells in the CNS and the lack of correlation with disease severity point toward other causes of neurological symptoms in humans. They are thought to be associated with cytokine storm, neuroimmune stimulation, or systemic SARS-CoV-2 spread [23], rather than extensive direct viral damage as seen in K18-hACE2 mice. Additional factors contributing to human CNS pathology may include pneumonia-related hypoxia, a hypercoagulable state, sepsis, or multiple organ failure may also contribute to CNS pathology [20]. The diffuse microgliosis observed in humans, especially in the brain stem and cerebellum [20,23], also corroborates this hypothesis and suggests systemic rather than focal microglial activation compared to K18-hACE2 mice.

However, despite the broader viral antigen dissemination in K18-hACE2 mice, both species share key histological features such as mononuclear meningoencephalitis and microgliosis, underscoring the complexity of SARS-CoV-2’s neurological impact across species. Nonetheless, the mechanistic sequence appears accelerated and intensified in this mouse model with (1) systemic cytokine priming, (2) early microglial activation, (3) direct viral invasion, (4) morphological transformation of microglia and lymphocyte infiltration.

### 4.4. Neurofilament Accumulation Points to Axonal Transport Dysfunction in the CNS

Neurofilaments (NF), composed of low-, medium-, and high-molecular weight subunits (NFL, NFM, NFH) and α-internexin, are intermediate filaments involved in maintaining neuronal structure and function [95]. These NFs are synthesized in the neuronal cell body and transported along axons, where they undergo degradation [95,96] and extensive phosphorylation [97,98]. The non-phosphorylated form (nNF) is typically present in the perikaryons and dendrites, while the phosphorylated form (pNF) predominantly resides in the axons [97,98]. Hence, the accumulation of nNF within the axons of the cerebral white matter observed in this study points to axonal dysfunction, associated either with defective NF subunit assembly, abnormal NF phosphorylation and/or degradation, or aberrant axonal transport [99,100,101,102]. In addition, the simultaneous absence of pNF accumulation supports the possibility of disturbance in NF phosphorylation, assembly, or degradation [103]. Abnormal accumulation of NFs has been associated with neurodegenerative disease in humans, such as Alzheimer’s disease, Parkinson’s disease, and amyotrophic lateral sclerosis [95,104]. Over and above, elevated levels of NF in plasma and cerebrospinal fluid have been correlated with severe disease progression and the onset of neurological symptoms in COVID-19 patients [105,106,107,108].

Temporal variability in marker sensitivity may also be relevant [109,110], as demonstrated in a pharmacologically induced murine demyelination model in which nNF-positive axonal bulbs were detected during the acute phase of disease, while β-APP and synaptophysin became more prominent at later time points [110]. In this study, other markers of axonal damage, including β-APP and pNF, and the motor protein kinesin, showed no significant differences between infected and control groups. Although both β-APP and NF rely on kinesin and the motor protein dynein for transport, axonal transport of β-APP is fast [111] while that of NF is slow [102].

Interestingly, multifocal aberrant accumulation of nNF in the brains of SARS-CoV-2-infected K18-hACE2 mice has not been reported so far. Moreover, it also represents an uncommon finding in COVID-19 patients and is usually associated with hypoxia and/or hemorrhage [83,112]. Of note, these accumulations are present within the white matter of the cerebellum, which usually shows only limited SARS-CoV-2-related alterations [12,26,82]. Considering the observed vacuolization of cerebellar white matter in a previous study [26], for which, however, no relevance in the context of SARS-CoV-2 infection could be confirmed, the nNF accumulations observed in this study also suggest degenerative processes in the cerebellum. These findings highlight the complexity of axonal dysfunction in SARS-CoV-2-infected mice and emphasize the need for further exploration of the underlying mechanisms.

### 4.5. SARS-CoV-2 Invades Ocular Tissues via Neuronal Routes in K18-hACE2 Mice

In this study, SARS-CoV-2 was detected in the retinas of K18-hACE2 mice, primarily in the retinal ganglion cell layer and in one animal in the inner nuclear cell layer. A similar distribution was observed in previous studies in which spike protein was present in retinal ganglion cells at 6 dpi [35], as well as iridocorneal sections and the ciliary body, in addition to the retina from 7 dpi to 21 dpi [36]. Likewise, viral antigen in this study peaked at 6 dpi and persisted through 7/8 dpi in some animals, suggesting delayed ocular infection. These findings parallel observations in humans, where SARS-CoV-2 has been detected in various ocular structures, including the retina [37,38,40]. However, while this study is showcasing the direct influence of the virus on ocular tissues, the indication of a systemic genesis for retinopathies, as observed both in animal models [36] and in humans [28,40,43], was not observed.

In the present study, microgliosis was present in the optic chiasm, suggesting neuroinflammation, a rare finding shared between animal models [35,36]. Similarly, in humans, optic neuritis has been described as a rare sequela of COVID-19 but appears to be more related to antibodies against myelin oligodendrocyte glycoprotein and aquaporin-4 [113,114,115]. However, pathomorphologic analysis in the present study revealed no indication of inflammatory processes in the retina. Conversely, other studies have described a variable thickness of the retina as well as an infiltration of lymphocytes and neutrophilic granulocytes [35]. Furthermore, an increase in chemokines, cytokines, as well as the expression of pattern-recognition receptors, could be demonstrated by molecular techniques [35,36].

Although viral presence was observed in the retina, the lack of or only mild detection of axonal damage markers and caspase 3 indicates only limited morphological neuronal damage, suggesting subclinical ocular involvement. Still, a substantial functional impairment cannot be excluded. These findings contrast with reports of severe damage in humans and animals, where retinal microvasculopathy and optic neuropathies have been observed [36,40,43,116,117]. Nonetheless, SARS-CoV-2 can infect multiple retinal cell types [36,40,42], potentially disrupting the functional integrity and contributing to retinal dysfunction during infection.

Together, in addition to the growing evidence that SARS-CoV-2 can enter eye tissue and induce inflammation via a neuronal pathway [35], these results mimic some of the clinical outcomes observed in human COVID-19 patients. Further research is essential to clarify the mechanisms of ocular infection, the extent of viral replication, and the long-term consequences of SARS-CoV-2 on vision.

Interestingly, this study observed no infection in the trigeminal nerve nor the retinal vessels, or other tissues examined. These findings contrast with earlier reports on the potential pathways for SARS-CoV-2 entry into the CNS and, subsequently, the eye in this animal model [35,36]. The use of different viral strains, despite the same animal model, may explain the discrepancies in tissue infection observed in this study. The tropism of the virus may vary between SARS-CoV-2 variants, as previously observed by mutations in the spike protein of newer variants, whose ability to infect certain tissues, such as the CNS, has been altered [118]. Some strains also exhibit reduced virulence [26,119], which could limit their ability to invade tissues like the trigeminal nerve or retina, as observed here. Differences in ACE2 receptor binding affinity may also play a role. Since SARS-CoV-2 uses ACE2 to enter host cells, mutations in the virus can affect its binding strength, potentially influencing which tissues are more susceptible to infection [120]. Over and above, some viral strains may be more readily recognized and neutralized by the host immune system, limiting their spread to tissues like the nervous system [121]. These factors likely contribute to the observed differences in tissue infection.

## 5. Conclusions

This study provides a comprehensive spatiotemporal analysis of SARS-CoV-2 infection in the K18-hACE2 mouse model, focusing on the nasal cavity, lungs, brain, and eyes. In the nose, the highest viral antigen load and inflammation were observed at 3 dpi, particularly in the respiratory epithelium, with transient mild to moderate lesions that diminished by 7/8 dpi. In the lungs, peak histopathological changes were observed at 6 and 7/8 dpi, with alveolar inflammation, pneumocyte infection, and sporadic hemorrhage, mirroring severe pulmonary pathology seen in human COVID-19. Together, these findings highlight a temporally coordinated sequence of organ involvement and support the utility of the K18-hACE2 mouse as a robust model for investigating the neuropathogenesis and extrapulmonary manifestations of SARS-CoV-2. In the brain, the virus showed a predominantly basal distribution with widespread intraneuronal SARS-CoV-2 antigen, lymphohistiocytic meningoencephalitis, microgliosis, T cell infiltration, and neuronal vacuolation, particularly peaking at 6 days post-infection. In addition, an aberrant accumulation of non-phosphorylated neurofilament was reported for the first time in this animal model. In the eyes, viral antigen was detected mainly in retinal ganglion cells at 6 and 7/8 dpi without associated histological lesions or significant glial activation, suggesting retinal infection occurs without overt inflammation.

## Data Availability

The datasets generated and/or analyzed during the current study are available from the corresponding authors upon reasonable request.

## Supplementary Materials

The following supporting information can be downloaded at: https://www.mdpi.com/article/10.3390/v17070963/s1, Figure S1: Neuronal infection and vacuolation in brains of SARS-CoV-2-infected K18-hACE2 mice; Table S1: Detailed information about immunohistochemical staining procedure; Table S2: Semiquantitative scoring system for evaluation of nasal lesions; Table S3: Semiquantitative scoring system for evaluation of brain lesions.
