# Supplementary material for: Spatiotemporal Characterization of Changes in the Respiratory Tract and the Nervous System, Including the Eyes in SARS-CoV-2-Infected K18-hACE2 Mice
Source: Viruses. 2025 Jul 9;17(7):963. doi: 10.3390/v17070963 (PMC12300873; doi:10.3390/v17070963)
Supplement: Supplementary file 1 [file viruses-17-00963-s001.zip › viruses-3702560-supplementary.pdf]

viruses

Supplementary material

Spatiotemporal Characterization of Changes in the Respiratory Tract and the Nervous System, Including the Eyes in SARS-CoV-2-Infected K18-hACE2 Mice

Malgorzata Rosiak [1,2,†], Tom Schreiner [1,2,†], Georg Beythien [1,2], Eva Leitzen [1,2], Anastasiya Ulianytska [1], Lisa Allnoch [1], Kathrin Becker [1], Lukas M. Michaely [1], Sandra Lockow [1], Sabrina Clever [3], Christian Meyer zu Natrup [3], Asisa Volz [2,3], Wolfgang Baumgärtner [1,*], Malgorzata Ciurkiewicz [1], Kirsten Hülskötter [1,‡] and Katharina M. Gregor [1,‡]

[1] Department of Pathology, University of Veterinary Medicine Hannover, 30159 Hannover, Germany
[2] Center for Systems Neuroscience, University of Veterinary Medicine Hannover Foundation, 30159 Hannover, Germany
[3] Institute of Virology, University of Veterinary Medicine Hannover, 30159 Hannover, Germany
* Correspondence: wolfgang.baumgaertner@tiho-hannover.de
† Equal first authors.
‡ Equal last authors.

Figure S1. Neuronal infection and vacuolation in brains of SARS-CoV-2-infected K18-hACE2 mice

Table S1. Detailed information about immunohistochemical staining procedure.

Table S2. Semiquantitative scoring system for evaluation of nasal lesions

Table S3. Semiquantitative scoring system for evaluation of brain lesions

 https://doi.org/10.3390/v17070963

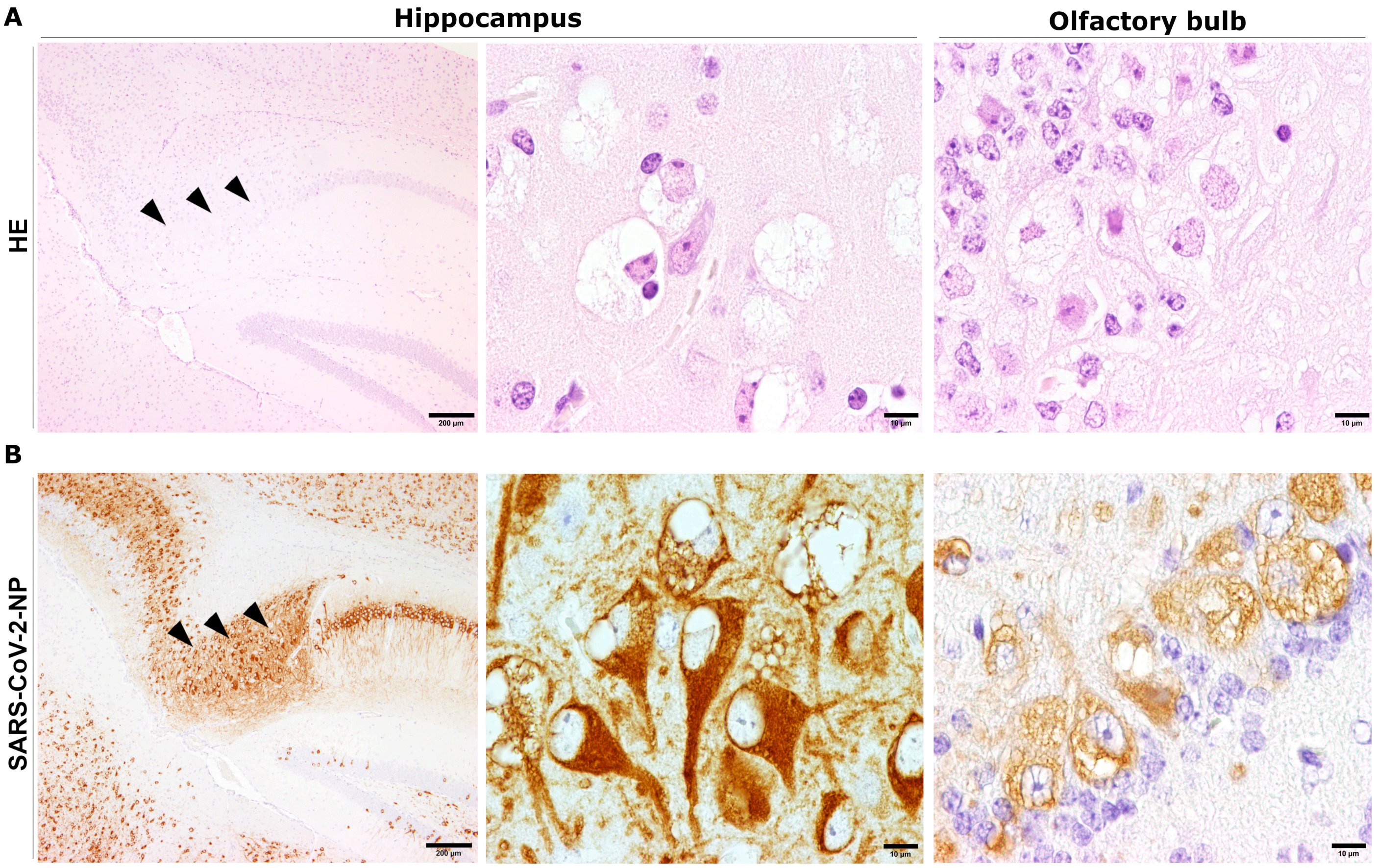

Figure S1. Neuronal infection and vacuolation in brains of SARS-CoV-2-infected K18-hACE2 mice. A) Representative sections of the hippocampus (left, low and high magnification), and olfactory bulb (right) of infected mouse at 6 dpi, stained with hematoxylin and eosin (HE) and **B)** SARS-CoV-2-NP immunohistochemistry, showing vacuolated cells with intracytoplasmic viral nucleoprotein antigen. Bar = 200 µm (low magnification); bar = 10 µm (high magnification).

Table S1. Detailed information about immunochistochemical staining procedure.

Primary antibody									
Specificity	Clonality	Source	Pretreatement	Blocking	Dilution				Secondary antibody
					Nose	Lung	Brain	Eye	
**ß₃-tubulin**	Polyclonal rabbit	PRB435 P, Covance	Simmering citrate buffer (pH 6)	Goat serum	1:4000	n.p.	n.p.	1:16000	GAR-b, BA1000, Vector
**ß-APP**	Monoclonal mouse	MAB348, Chemion	Simmering citrate buffer (pH 6)	None	n.p.	n.p.	1:4000	n.p.	EnVision mouse, Dako
**Caspase 3 (Asp175)**	Polyclonal rabbit	9661, Cell Signaling Technology	Simmering citrate buffer (pH 6)	Goat serum	n.p.	n.p.	n.p.	1:200	GAR-b, BA1000, Vector
**CD3**	Polyclonal rabbit	A0452, Dako	Simmering citrate buffer (pH 6)	Goat serum	n.p.	n.p.	1:200	1:200	GAR-b, BA1000, Vector
**CD45R/B220**	Monoclonal rat	553086BD, Biosciences	Simmering citrate buffer (pH 6)	None	n.p.	n.p.	1:2000	n.p.	-
**GFAP**	Polyclonal rabbit	Z0334, Dako	None	Goat serum	n.p.	n.p.	1:1000	1:1000	GAR-b, BA1000, Vector
**GS**	Polyclonal rabbit	PA528940, invitrogen	None	Goat serum	n.p.	n.p.	n.p.	1:400	GAR-b, BA1000, Vector
**Iba1**	Polyclonal rabbit	019-197; FUJIFILM Wako Pure Chemical Corp.	Simmering citrate buffer (pH 6)	Goat serum	n.p.	n.p.	1:8000	1:8000	GAR-b, BA1000, Vector
**Kinesin**	Polyclonal rabbit	R4505, Sigma	Simmering citrate buffer (pH 6)	Goat serum	n.p.	n.p.	1:2000	n.p.	GAR-b, BA1000, Vector
**MPO**	Polyclonal rabbit	ab9535, Abcam	Simmering citrate buffer (pH 6)	Goat serum	n.p.	n.p.	1:100	n.p.	GAR-b, BA1000, Vector
**nNF**	Monoclonal mouse	SMI-311R, Sternberger	Simmering citrate buffer (pH 6)	Goat serum	n.p.	n.p.	1:8.000	1:1000	GAM-b, BA9200, Vector
**pNF**	Monoclonal mouse	SMI-312, Biolegend	None	Goat serum	n.p.	n.p.	n.p.	1:8000	GAM-b, BA9200, Vector
**SARS-CoV-2-NP**	Monoclonal mouse	40143-MM05, Sino Biological	Simmering citrate-Na_2H_2EDTA buffer (pH 6.2)	-	1:16000	n.p.	1:16000	1:16000	-
**SARS-CoV-2-NP**	Polyclonal rabbit	40143-R019, Sino Biological	Simmering citrate buffer (pH 6)	-	n.p.	1:2000	n.p.	n.p.	-

Abbreviations: ß-APP, beta-amyloid precursor protein;GAM-b, biotinylated goat-anti-mouse IgG; GAR-b, biotinylated goat-anti-rabbit IgG; GFAP, glial fibrillary acidic protein; GS, glutamine synthetase; Iba1, ionized calcium-binding adapter molecule 1; kinesin, motor protein kinesin; MPO, myeloperoxidase; nNF, non-phosphorylated neurofilament; n.p., not performed; pNF, phosphorylated neurofilament; SARS-CoV-1-NP, nucleocapsid protein.

Table S2. Semiquantitative scoring system for evaluation of nasal lesions

**1.1. Extent of inflammation for respiratory and olfactory epithelium**		
0	No - minimal	Single inflammatory cells
1	Mild	1-2 layers of inflammatory cells
2	Moderate	3-4 layers of inflammatory cells
3	Severe	5-6 layers of inflammatory cells
4	Subtotal	≥7 layers of inflammatory cells
**1.2. Distribution of inflammation for respiratory and olfactory epithelium (percentage refers to total area of the respective compartment)**		
0	No - minimal	No inflammation to occasional foci with few inflammatory cells but overall less than 1% of assessable tissue
1	Mild	2-25% affected
2	Moderate	26-50% affected
3	Severe	51-75% affected
4	Subtotal	>75% affected
**1.3. Epithelial necrosis for respiratory and olfactory epithelium**		
0	No - minimal	Not observed
1	Mild	2-25% affected
2	Moderate	26-50% affected
3	Severe	51-75% affected
4	Subtotal	>75% affected
**1.4. Hyperplastic epithelium for respiratory and olfactory epithelium**		
0	No	
1	Yes	
**1.5. Total respiratory and olfactory epithelium scores**		
0-21 each	(extent of inflammation x inflammation distribution) + epithelial necrosis + hyperplastic epithelium	
**2. Intraluminal exudate**		
0	No	Not observed to less than 1 % of lumina affected
1	Mild	2-25% of lumina affected
2	Moderate	26-50% of lumina affected
3	Severe	51-75% of lumina affected
4	Subtotal	>75% of lumina affected
**3. Vasculopathy**		
0	No	Not observed to less than 1 % of vessels affected
1	Mild	2-25% of vessels affected
2	Moderate	26-50% of vessels affected
3	Severe	51-75% of vessels affected
4	Subtotal	>75% of vessels affected
**4. Total nasal score**		
0-50	Respiratory epithelium score + olfactory epithelium score + intraluminal exudate + vasculopathy	

Table S3. Semiquantitative scoring system for evaluation of brain lesions

**1. Extent of perivascular inflammation**		
0	No	No inflammation
1	Mild	Single perivascular inflammatory infiltrates
2	Moderate	2-3 layers of perivascular infiltrates
3	Severe	>3 layers of perivascular infiltrates
**2. Distribution of perivascular inflammation**		
0	No	No inflammation
1	Mild	Multifocal vessels (<50%)
2	Moderate	Multifocal vessels (50-75%)
3	Severe	Multifocal vessels (>75%)
**3. Vasculitis**		
0		No
1		Yes
**4. Cell death in vessel wall/perivascular space**		
0		No
1		Yes
**5. Vascular score**		
0-11		(perivascular inflammation extent x perivascular inflammation distribution) + vasculitis + cell death in vessel wall / perivascular space
